# Therapeutic Efficacy of Third-Generation Percutaneous Vertebral Augmentation System (PVAS) in Osteoporotic Vertebral Compression Fractures (OVCFs): A Systematic Review and Meta-analysis

**DOI:** 10.1155/2022/9637831

**Published:** 2022-05-07

**Authors:** Chunke Dong, Yuting Zhu, Jun Zhou, Liang Dong

**Affiliations:** ^1^Beijing Hospital of Traditional Chinese Medicine, Capital Medical University, Beijing, China; ^2^Beijing Tongzhou Integrative Medicine Hospital, Beijing, China; ^3^Beijing University of Chinese Medicine, Beijing, China; ^4^Department of Spine Surgery, Honghui Hospital, Xi'an Jiaotong University, No 555, YouYi East road, Xi'an, China

## Abstract

**Purpose:**

This study aimed to assess whether the third-generation PVAS was superior to percutaneous vertebroplasty (PVP) or percutaneous kyphoplasty (PKP) in treating patients with OVCFs.

**Methods:**

Databases, including Pubmed, Embase, and Cochrane library, were searched to identify relevant interventional and observational articles in vivo or in vitro comparing the third-generation PVAS to PVP/PKP in OVCFs patients. A meta-analysis was performed under the guidelines of the Cochrane Reviewer's Handbook.

**Results:**

11 in vivo articles involving 1035 patients with 1320 segments of diseased vertebral bodies and 8 in vitro studies enrolling 40 specimens with 202 vertebral bodies were identified. The vivo studies indicated no significant differences were found in visual analog scale (VAS), Oswestry Disability Index (ODI), operation time, or injected cement volume (*P* > 0.05). The third-generation PVAS was associated with significant improvement in vertebral height and Cobb angle (*P* < 0.05) and also with a significantly lower risk of cement leakages and new fractures (*P* < 0.05). The vitro studies suggest that the third-generation PVAS was associated with better anterior vertebral height (AVH) and kyphotic angle (KA) after deflation and cement. No significant differences were found in stiffness or failure load after cement between the two groups (*P* > 0.05).

**Conclusion:**

Based on current evidence, although providing similar improvement in VAS and ODI, the third-generation PVAS may be superior to PVP/PKP in local kyphosis correction, vertebral height maintenance, and adverse events reduction. Further high-quality randomized studies are required to confirm these results.

## 1. Introduction

Over the past few decades, as a minimally invasive procedure, the PVAS has been considered the optimal management for symptomatic OVCFs [1–3]. PVP, the first-generation PVAS, can provide effective and rapid pain relief and spinal stabilization via direct injecting polymethylmethacrylate (PMMA) into the inter-trabecular marrow space of a fractured vertebra [4]. However, this procedure is challenging to restore vertebral height and with up to 54.7% of cement leakage [5]. The matters lead to the evolution of the second-generation PVAS, PVP, which can correct kyphosis through inflation of a balloon inside the collapsed vertebral body [6]. The balloon could also create a cavity, allowing more viscous cement to be injected with lower pressure, thereby significantly reducing leakage risk [7]. Notwithstanding, PKP has been proved to be associated with a higher rate of refracture on cemented vertebrae than PVP [8], especially with an intravertebral cleft (IVC) [9–12]. Moreover, secondary loss of the initial reduction may occur after balloon deflation [13].

These concerns promote the emergence of the third-generation PVAS. This novel expandable scaffolding device is permanently implanted into the vertebral body to restore reduction mechanically before injecting bone cement. Up to now, several systems are available: SpineJack® [14–17], Vertebral Body Stenting® (VBS) [18–20], OsseoFix® System [21, 22], and Kiva® System [23–25]. In theory, the third-generation PVAS is superior to PVP/PKP in height restoration and height maintenance. However, inconsistent results were obtained from different trials comparing clinical symptoms recovery, vertebral height restoration, and adverse events of the third-generation PVAS versus PVP/PKP in patients with OVCFs [20, 24, 26]. In order to provide more evidence for clinical decision-making, we conducted a systematic review and meta-analysis to integrate existing evidence from relevant in vivo or in vitro trials to evaluate the superiority of third-generation PVAS over PVP/PKP in the treatment of patients with OVCFs.

## 2. Methods

### 2.1. Search Strategy

This systematic review and meta-analysis was conducted based on the Preferred Reporting Items for Systematic Reviews and Meta-analyses (PRISMA) statement [27]. The current systematic review protocol was registered on INPLASY.COM (ID: INPLASY202110015) and available in full https://inplasy.com/inplasy-2021-1-0015/. A systematic computer-based retrieval for all relevant published articles in vivo or in vitro was performed in medical databases including Pubmed, Embase, and Cochrane Library from inception to December 31, 2020. The search terms for the study object: “Spinal Fractures [Mesh]” OR^“^Spinal Fracture∗^”^ OR^“^thoracic fracture∗^”^ OR^“^lumbar fracture∗^”^ OR^“^vertebral fracture∗.^”^ The intervention's search terms are as follow: “KIVA” OR “SpineJack” OR “vertebral body stent∗” OR “Stentoplasty” OR “VBS” OR “OsseoFix.” We also checked the reference lists of all including articles to avoid any initially omitted studies. There was no publication language and population limitation during the systematic review. A detailed list of search strategies could be found in Supplemental Appendix 1.

### 2.2. Inclusion and Exclusion Criteria

Trials eligible for inclusion in this meta-analysis were as follows: (1) interventional studies (RCTs) and observational studies (cohort or case-control studies) in vivo or in vitro; (2) clinical or cadaveric studies compared the efficacy of third-generation PVAS (SpineJack, KIVA, VBS, or OsseoFix) with PVP or PKP for OVCFs; and (3) studies reported at least one outcome of interest: VAS, ODI, KA, Cobb angle, AVH, midline vertebral height (MVH), posterior vertebral height (PVH), injected cement volume, cement leakage, or adjacent vertebral fracture. Exclusion criteria: (1) Pathological fractures due to primary or metastatic tumors, infection, or tuberculosis; (2) Non-original articles (case reports, reviews, letters, meta-analyses, conference abstract, and editorials).

### 2.3. Selection Criteria

D. CK. and Z. YT. independently screened eligible studies based on the criteria mentioned above. Firstly, the titles and abstracts were reviewed to exclude articles that obviously did not meet the inclusion criteria. Then, a full-text review was conducted to ensure met all the inclusion criteria. All disagreements were resolved by reaching a consensus among the researchers.

### 2.4. Data Extraction and Quality Assessment

Two investigators (D. CK. and Z. YT.) independently extracted the following characteristics from included studies: author, publication year, country, study design, interventions, and patient or human cadaveric information (age, gender, BMD, and sample size). Data forms were converted according to the Cochrane Handbook [28], and figure data was extracted by manual measurement. The methodological quality of the RCTs and no-RCTs (cohort or case-control studies) was assessed independently by D. CK. and W. HY. using the Cochrane Collaboration's Risk of Bias Tool [29] and Newcastle-Ottawa scale (NOS) [30], respectively. Any discrepancies of data extraction and quality assessment were settled by discussing a third independent author (Z.J.).

### 2.5. Data Analysis

This meta-analysis was conducted with Review Manager 5.3 software (Cochrane Collaboration, Oxford, UK). Continuous data were calculated through the mean difference (MD) or standardized mean difference (SMD) with 95% CI. We calculated risk ratio (RR) with 95% CI to evaluate the cement leakage and adjacent level fractures. Heterogeneity across studies was assessed using Cochran's Q and *I*^2^ statistics, and *P* < 0.1 and I^2^ > 50% were considered statistical heterogeneity [31]. A fixed-effects model was conducted when I^2^ ≤ 50%; otherwise, a random-effects model was performed. Publication bias was assessed statistically by Stata 12.0 (Begg and Egger tests). Sensitivity analysis was also introduced to detect the result's stability. *P* < 0.05 was considered statistically significant.

### 2.6. Search Results

**Figure 1:**
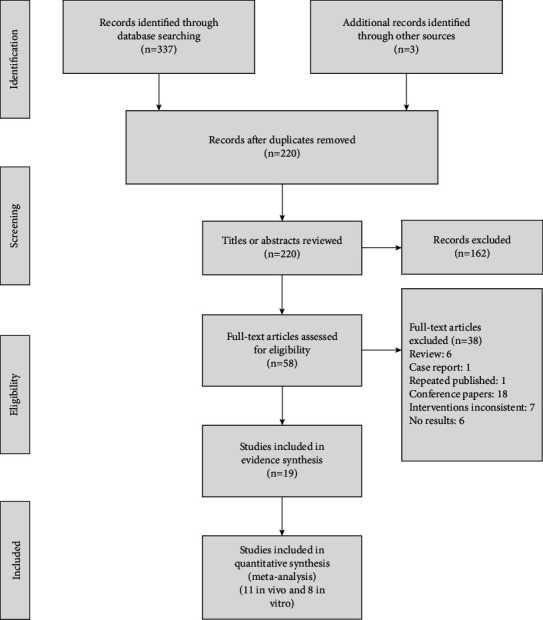
Summary of study selection and inclusion process.

The comprehensive search initially identified a total of 340 potential articles (PubMed 128, Embase 172, the Cochrane Library 37, and additional in the reference lists 3), in which 120 duplicates were removed. After screening the titles and abstracts, 58 full-text articles were assessed in more detail for eligibility. After excluding 6 reviews, 1 case report, 1 repeated published, 18 conference papers, 7 interventions inconsistent, and 6 no results, 11 in vivo [14, 18–20, 23–26, 32–34] and 8 in vitro [21, 22, 35–40] studies were included in this study (Figure 1).

### 2.7. Study Characteristics

The in vivo studies included 5 RCTs [14, 20, 23, 25, 26] and 6 retrospective cohort studies [18, 19, 24, 32–34] involving 1035 patients with 1320 segments of diseased vertebral bodies. Among them, four trials [14, 26, 33, 34] compared SpineJack with PVP or PKP, while four [18–20, 32] compared VBS with PVP or PKP, and three [23–25] compared KIVA versus PKP. The in vitro studies consist of 5 RCTs [21, 22, 35, 37, 38] and 3 prospective cohort studies [36, 39, 40], with a resulting count of 40 specimens and 202 vertebral bodies. In the experimental group, two studies [35, 38] used SpineJack, three [36, 39, 40] used VBS, two [21, 22] used OsseoFix, and only one [37] used Kiva. All control groups were treated with PKP. The detailed characteristics of the involved in vivo and in vitro studies are summarized in Tables 1 and 2, respectively.

### 2.8. Quality of Included Studies

**Figure 2:**
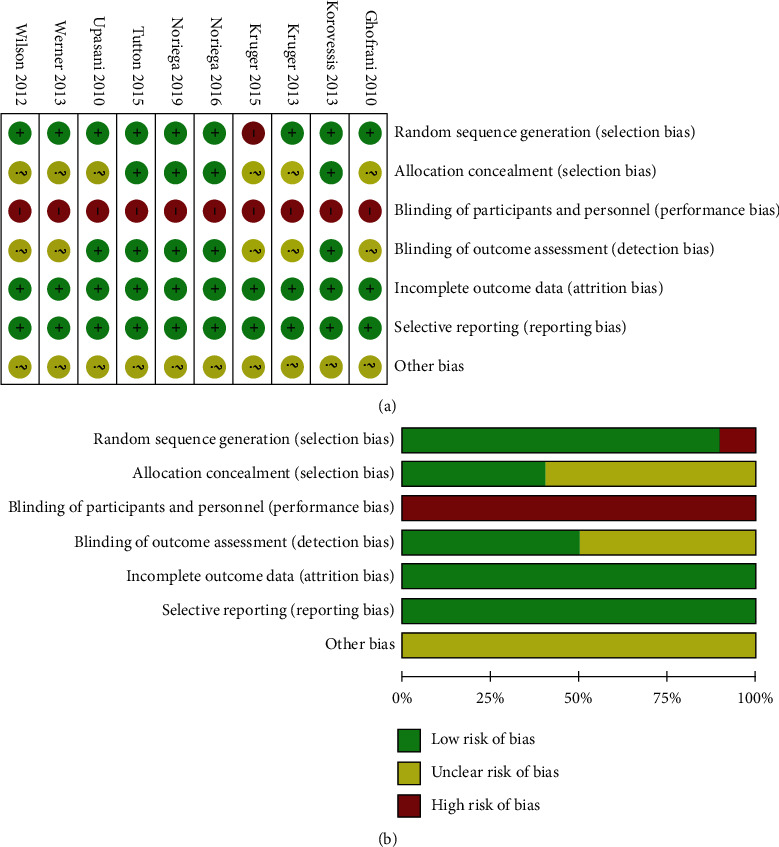
The methodological quality of RCTs: risk of bias summary (a) and risk of bias graph (b).

The risk of bias of the included 10 RCTs was used the Cochrane Collaboration's Tool, as shown in Figure 2. The random sequence generation was low risk in nine studies [14, 20–23, 25, 26, 37, 38], and the illustration of allocation concealment was unclear for 6 trials [20–22, 35, 37, 38]. The blinding of researcher was evaluated as “high risk” for all 10 studies [14, 20–23, 25, 26, 35, 37, 38], and the blinding of outcome was unclear for 5 trials [20, 22, 35, 37, 38]. 9 cohort studies were appraised according to the NOS in which 3 studies [36, 39, 40] assigned 9 scores, 3 studies [19, 33, 34] assigned 8 scores, and 2 studies [18, 32] assigned 7 scores were considered high quality. One study [24] given 6 scores was regarded as moderate quality.

## 3. Meta-analysis of In Vivo Studies

### 3.1. Pooled Analysis of VAS and ODI

**Figure 3:**
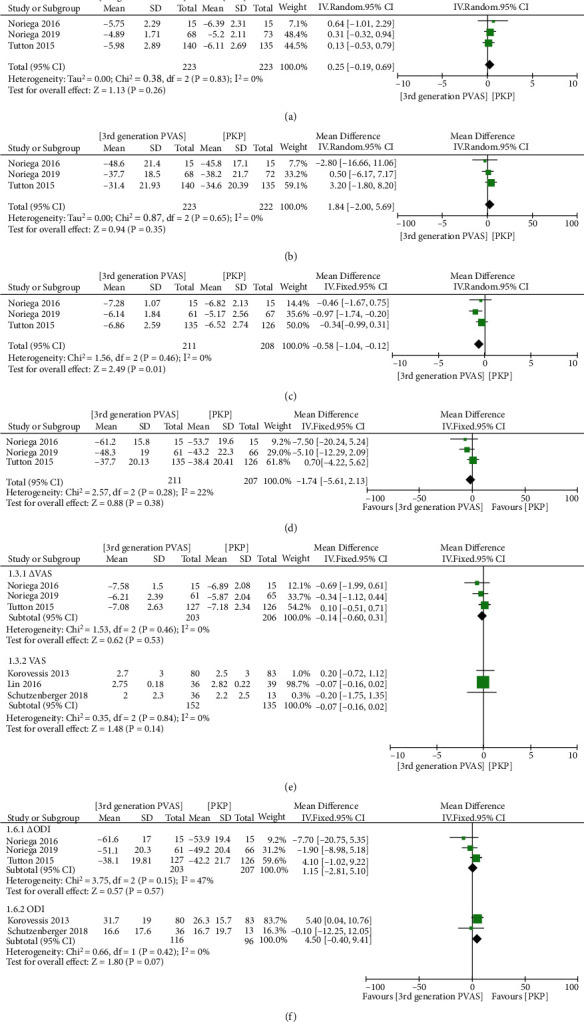
Forest plot and pooled data of short-term △VAS (a) and △ODI (b), mid-term △VAS (c) and △ODI (d), long-term △VAS (e) and △ODI (f), and long-term VAS (g) and ODI (h) between the two groups in vivo studies.

We divided the results into short-term (≤1 month), mid-term (3 ~ 6 months), and long-term (≥12 months). 3 studies [14, 23, 26] on 446 patients reported the short-term and mid-term △VAS and △ODI. No significant difference was found in short-term △VAS and △ODI between the 2 groups (MD = 0.25, 95% CI -0.19 to 0.69, *P* = 0.26, Figure 3(a); MD = 1.84, 95% CI -2.00 to 5.69, *P* = 0.35, Figure 3(b), respectively). The overall effect also showed no significant difference in mid-term △ODI (MD = −1.74; 95% CI -5.61 to 2.13; *P* = 0.38; Figure 3(d)), whereas the result indicated that the third-generation PVAS had significantly better improvement in mid-term △VAS than the PKP (MD = −0.58; 95% CI, -0.99 to 0.31; *P* = 0.01; Figure 3(c)). Three studies [14, 23, 26] recorded long-term △VAS and △ODI, the other three [19, 25, 34] recorded long-term VAS, and two [19, 25] recorded long-term ODI. The summarized estimate of effect size revealed no significant differences in long-term results between the two groups (MD_△VAS_ = −0.14, 95% CI -0.60 to 0.31, *P* = 0.53, Figure 3(e); MD_VAS_ = −0.07; 95% CI -0.16 to 0.02, *P* = 0.84, Figure 3(e); MD_△ODI_ = 1.15, 95% CI -2.81 to 5.10, *P* = 0.57, Figure 3(f); MD_ODI_ = 4.50, 95% CI -0.40 to 9.41, *P* = 0.07, Figure 3(f), respectively).

#### 3.1.1. Pooled Analysis of Vertebral Height and Cobb

**Figure 4:**
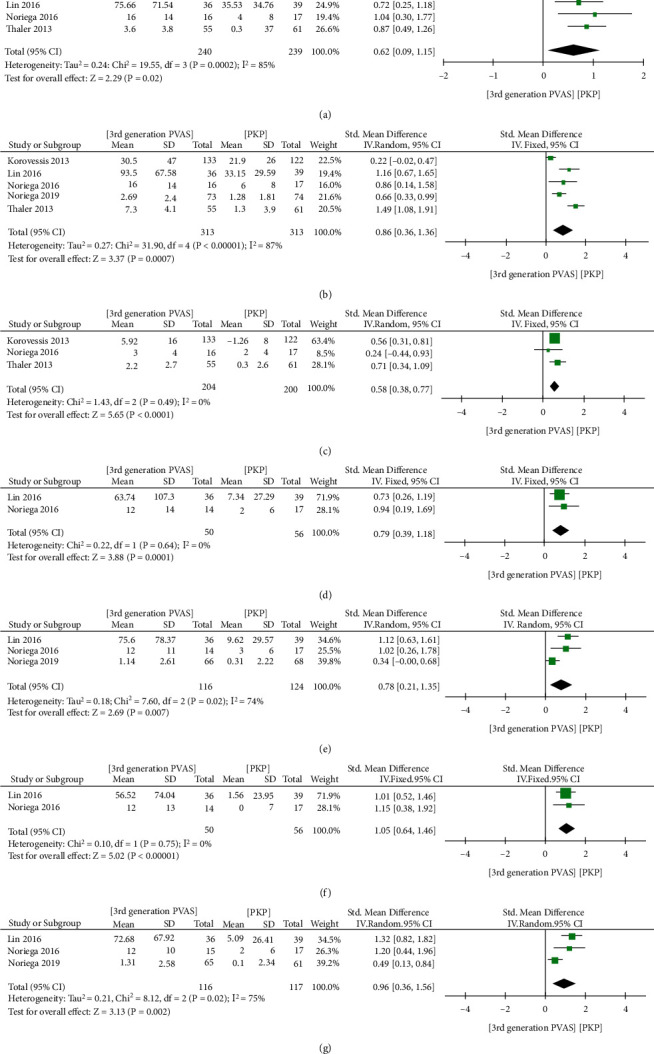
Forest plot and pooled data of short-term AVH (a), MVH (b), PVH (c), mid-term AVH (d), MVH (e), and long-term AVH (f) and MVH (g) between the two groups in vivo studies.

Four trials [14, 18, 25, 34] were involved in analyzing short-term AVH, five [14, 18, 25, 26, 34] for short-term MVH, and three [14, 18, 25] for short-term MVH. There were two articles [14, 34] provided data for mid- and long- term AVH and three [14, 26, 34] for mid- and long- term MVH.  Figure 4 illustrates significant improvements in short-term AVH (SMD = 0.62, 95% CI 0.09 to 1.15, *P* = 0.02, Figure 4(a)), MVH (SMD = 0.86, 95% CI 0.36 to 1.36, *P* = 0.0007, Figure 4(b)), PVH (SMD = 0.58, 95% CI 0.38 to 0.77, *P* < 0.00001, Figure 4(c)), mid-term AVH (SMD = 0.79, 95% CI 0.39 to 1.18, *P* = 0.0001, Figure 4(d)), MVH (SMD = 0.78, 95% CI 0.21 to 1.35, *P* = 0.007, Figure 4(e)), and long-term AVH (SMD = 1.05, 95% CI 0.64 to 1.46, *P* < 0.00001, Figure 4(f)) and MVH (SMD = 0.96, 95% CI 0.36 to 1.56, *P* = 0.002, Figure 4(g)).

**Figure 5:**
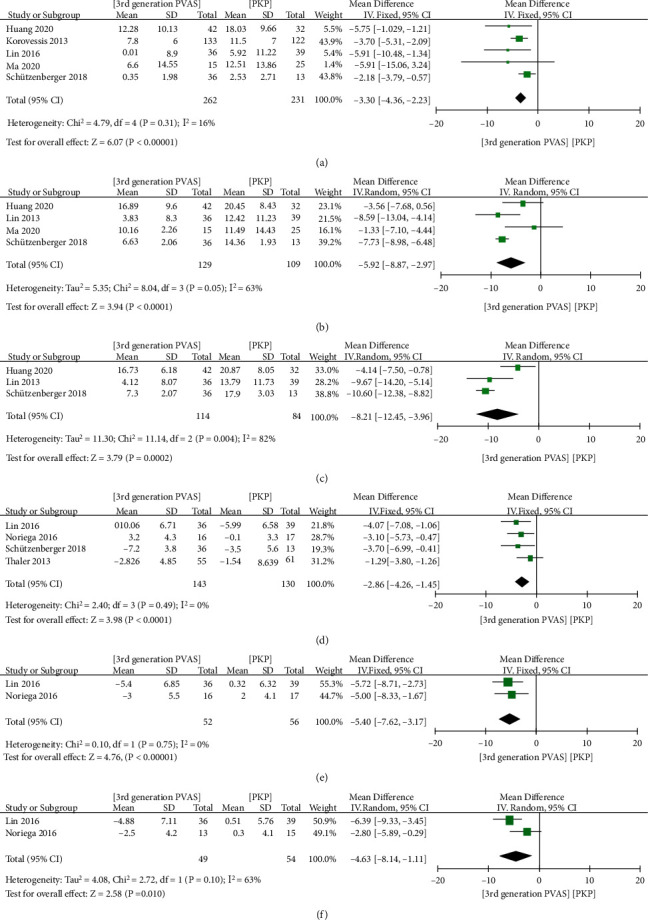
Forest plot and pooled data of short- (a), mid- (b), long-term (c) Cobb and short- (e), mid- (f), and long-term (g) △Cobb between the two groups in vivo studies.

Furthermore, the third-generation PVAS was associated with significant improvement in Cobb (short-term: MD = −3.30, 95% CI -4.36 to -2.23, *P* < 0.00001, Figure 5(a); mid-term: MD = −5.92, 95% CI -8.87 to -2.97, *P* < 0.0001, Figure 5(b); long-term: MD = −8.21, 95% CI -12.45 to -3.96, *P* = 0.0002, Figure 5(c)) and △Cobb (short-term: MD = −2.86, 95% CI -4.26 to -1.45, *P* < 0.0001, Figure 5(d); mid-term: MD = −5.40, 95% CI -7.62 to -3.17, *P* < 0.00001, Figure 5(e); and long-term: MD = −4.63, 95% CI -8.14 to -1.11, *P* = 0.010, Figure 5(f)).

#### 3.1.2. Pooled Analysis of Operation Time and Injected Cement Volume

**Figure 6:**
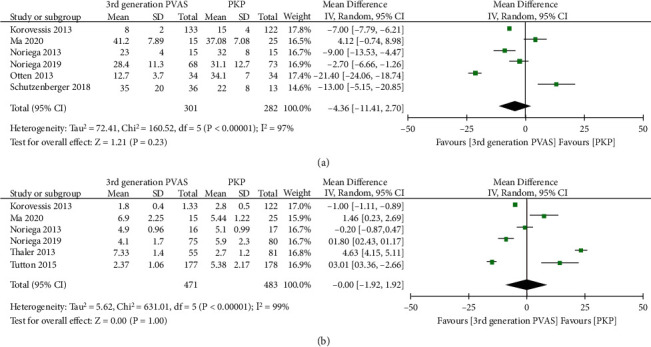
Forest plot and pooled data of operation time (a) and injected cement volume (b) between the two groups in vivo studies.

The data of the operation time were available for six studies [14, 19, 24–26, 32]. The random-effect was employed due to the significant heterogeneity between the studies (*P* < 0.00001, *I*^2^ = 97%). The pooled analysis declared that no significant difference between the two groups (MD = −4.36, 95% CI -11.41 to 2.70, *P* = 0.23, Figure 6(a)). Six trials [14, 18, 23, 25, 26, 32] included have compared the bone cement injected between the two procedures. The pooled analysis of a random-effects model indicated that the amount of bone cement injected was similar in the two groups (MD = −0.00, 95% CI -1.92 to 1.92, *P* = 1.00, Figure 6(b)).

The sensitivity analysis was performed by omitting one study in each round to examine the impact on the overall result. The operation time in the third-generation PVAS group was not significantly different from that in the PKP group when omitting any of the studies except Schützenberger et al. [19]. In addition, the sensitivity analysis suggested no significant variation in bone cement injected attributable to heterogeneity.

#### 3.1.3. Pooled Analysis of Adverse Events

**Figure 7:**
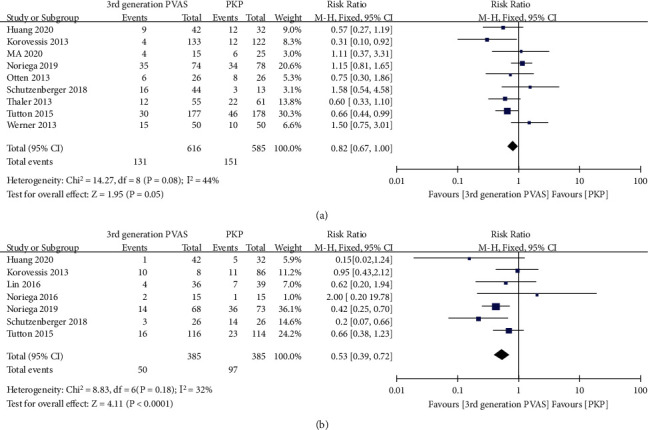
Forest plot and pooled data of adverse events in vivo studies: bone cement leakage (a) and new fractures (b).

Adverse events related to bone cement leakage were reported in nine studies [18–20, 23–26, 32, 33], with a total of 1201 injured vertebra bodies (616 in the intervention group and 585 in the control group). Overall, the summarized estimate of effect size indicated a slightly significant difference between the two groups (RR = 0.82, 95% CI 0.67 to 1.00, *P* = 0.05, Figure 7(a)), which favored the third-generation PVAS, with moderate heterogeneity (*I*^2^ = 44%, *P* = 0.08).

Seven studies offered relevant data on new fractures between the two groups. The comprehensive meta-analysis estimated a borderline statistically significant RR of 0.52 (95% CI 0.39 to 0.72; *P* < 0.0001), suggesting a lower risk of new fractures with the third-generation PVAS (Figure 7(b)). The *I*^2^ value attributed 32% variation to heterogeneity; therefore, a fixed-effects model was used.

The Begg and Egger tests indicated no evidence of publication bias for bone cement leakage (*P* = 0.754 and 0.659, respectively) and new fractures (*P* = 0.764 and 0.914, respectively) in terms of the 11 articles in vivo.

## 4. Meta-analysis of In Vitro Studies

### 4.1. Pooled Analysis of AVH and KA

**Figure 8:**
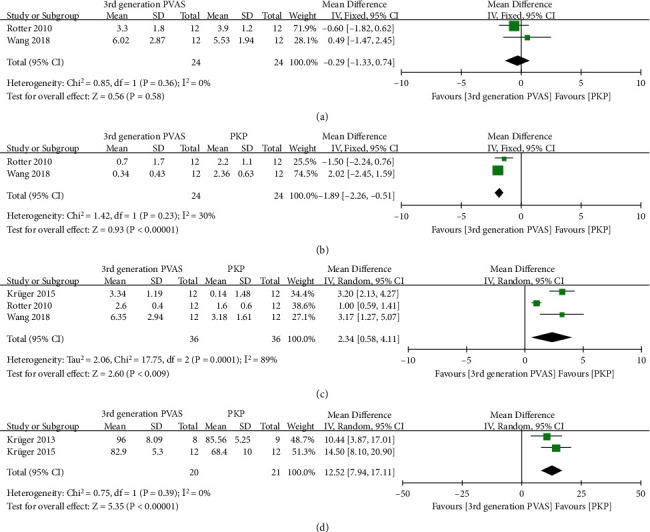
Forest plot and pooled data of AVH gain after reposition (a), loss of AVH after deflation (b), AVH gain (c), and AVH% (d) after cement in vitro studies.

AVH changes after reposition and deflation were recorded in two studies [36, 40], changes after cement in three [35, 36, 40], and the final AVH% after cement in the other two [35, 38]. The overall pooled analysis suggest no significant difference in AVH gain after reposition between the two groups (MD = −0.29, 95% CI -1.31 to 0.74, *P* = 0.58, Figure 8(a)). In contrast, the loss of AVH in the third-generation PVAS group after deflation was significantly less than the PKP group (MD = −1.89, 95% CI -2.26 to -1.51, *P* < 0.00001, Figure 8(b)). The pooled analysis showed that the third-generation PVAS was associated with better AVH gain and final AVH% after cement when compared with PKP (MD = 2.34, 95% CI 0.58 to 4.11, *P* = 0.009, Figure 8(c); MD = 12.52, 95% CI 7.94 to 17.11, *P* < 0.00001, Figure 8(d), respectively).

**Figure 9:**
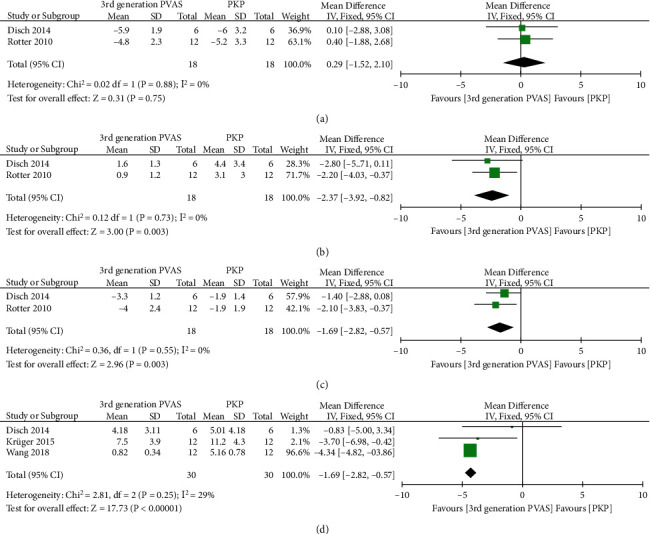
Forest plot and pooled data of △KA after reposition (a), △KA after deflation (b), △KA (c), and KA (d) after cement in vitro studies.

KA changes after reposition, deflation, and cement were described in two studies [36, 39] and the final KA after cement in the other three [35, 39, 40]. The pooled analysis indicated the absence of significant differences in △KA after reposition between the two groups (MD = 0.29, 95% CI -1.52 to 2.10, *P* = 0.75, Figure 9(a)). In contrast, the loss of KA was significantly smaller in the third-generation PVAS after deflation (MD = −2.37, 95% CI -3.92 to -0.82, *P* = 0.003, Figure 9(b)). After cement, the final △KA and KA were also significantly smaller in the third-generation PVAS compared to the PKP (MD = −1.69, 95% CI -2.82 to -0.57, *P* = 0.003, Figure 9(c); MD = −4.28, 95% CI -4.75 to -3.81, *P* < 0.00001, Figure 9(d), respectively).

### 4.2. Pooled Analysis of Stiffness and Failure Load after Cement

**Figure 10:**
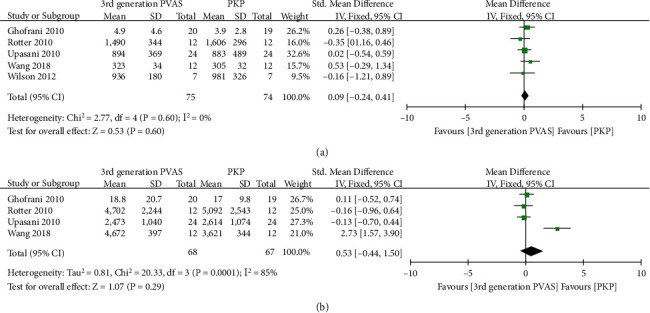
Forest plot and pooled data of stiffness (a) and failure load (b) after cement in vitro studies.

Adequate data on stiffness after cement was present in five studies [21, 22, 36, 37, 40], and the difference in overall estimate was not statistically significant (SMD = 0.09, 95% CI -0.24 to 0.41, *P* = 0.60, Figure 10(a)). The data of failure load was available for four trials [21, 22, 36, 40]. The pooled results demonstrated no significant difference between the two groups (SMD = 0.53, 95% CI -0.44 to 1.50, *P* = 0.29, Figure 10(b)).

The sensitivity analysis of failure load indicated no significant impact on the final pooled result following when omitting any of the eligible studies. The results of the Begg and Egger tests in stiffness and failure load also proved the absence of significant publication bias in terms of the 8 articles in vitro (*P* > 0.05).

## 5. Discussion

As the aging process accelerates, OVCFs contribute to a major health problem worldwide due to the loss of health-related quality of life and high healthcare costs [41]. Treatment options usually contain conservative management (analgesics, bracing, bed rest, and physical therapy) and minimally invasive surgery (PVP and PKP). Although most studies suggested PVP and PKP appear to be associated with longer post-discharge survival rates and a cost-effective alternative to nonoperative management [2, 41–43], two high-quality RCTs [44, 45] indicated that patients could not benefit from vertebral augmentation in resolving pain and disability. Furthermore, refractures and new fractures, the most severe complications, were not avoided after PVP or PKP. Currently, various forms of third-generation PVAS involving SpineJack, KIVA, VBS, and OsseoFix have been evaluated by cadaver and clinical studies [15, 20, 25, 37, 39]. However, it is still questionable whether the third-generation PVAS is superior to PKP or PVP. As far as we know, our study is the first systematic review and meta-analysis to comprehensively compare the efficiency of the third-generation PVAS versus PVP/PKP for OVCFs in vitro and in vivo.

The application of the third-generation PVAS in OVCFs has expanded enormously during the last decade [46–50]. Although the facilities of each system are different, all of them are characterized by implanting permanent expandable devices to hydraulically or mechanically control reduction of the vertebral fracture and the sagittal balance of the spine [48, 49]. Due to the lack of mechanical reduction ability of PVP, the recovery of vertebral height depends on intraoperative posture or the use of stents to induce scoliosis. PKP can restore vertebral height utilizing a balloon dilatation; nevertheless, it is difficult to maintain height after balloon deflation, even in a lordotic position, where an approximately 110 N compression is still imposed on the vertebrae, resulting in the collapse of the created cavity [36].

Concerning in vitro experiments, our meta-analysis indicated that sagittal height restoration and kyphosis correction were significantly better when using the third-generation PVAS than PKP. The correction loss of PKP may attribute to the deflation effect: The created cavity may collapse after balloon deflation, before cement augmentation, due to the existing constant preload exerted on the vertebra even in a lordotic position [35]. The third-generation PVAS, as specific mechanical properties permanent implant devices, can provide the immediate intraoperative load-bearing capability to offset the deflation effect before bone cement injection [36]. Wang et al. [40] found that the VBS could withstand a compressive load of 226 N, exceeding the existing preload of 110 N which exerted on the vertebrae. Despite the lack of accurate values for other devices in included studies, we believe that the effects are similar. No significant differences were found in failure load and stiffness after cement augmentation between the two groups in our meta-analysis, which demonstrated that the implanted permanent expandable devices did not affect the biomechanical behavior of the treated vertebral body.

Without considering the influence of surrounding soft tissue, in vitro studies simulate the conditions of immediate post-operation and offer a promising result for the third-generation PVAS, whereas the effects of healing and gradual restoration of activity cannot be evaluated. Thus, we also compared the radiological and clinical results of the third-generation PVAS and PVP/PKP in clinical studies in our systems review. Similar to cadaver studies, experimental results in clinical indicate that the third-generation PVAS was more effective in restoring vertebral body height and correcting kyphosis angle than PVP/PKP at all time points. On the contrary, no significant differences were observed in terms of short-, mid-, and long-term VAS and ODI, except the mid-term VAS. Previous meta-analysis [7, 51] had found that painful and functional improvement were positive correlated with vertebral height recovery and kyphosis correction after PKP/PVP for OVCFs, which is not consistent with our research. To date, it could not yet be established with certainty that height gain and improved outcomes in pain relief and quality of life are clinically relevant. Crucially, the common denominator for pain relief after the third-generation PVAS or PKP/PVP is the internal cement splint [52]. In addition, the surrounding ligaments, muscles and osteoporosis could also affect the outcomes.

Treatment-associated complications, such as cement leakage and new fractures, have caused widespread concern among surgeons. Our results illustrate that the third-generation PVAS could lower the risk of cement leakage and refracture compared to PKP/PVP. It is generally accepted that the high-pressure injection of low-viscosity bone cement would lead to a higher risk of cement leakage [7, 53]. Just like PKP, the third-generation PVAS can create a cavity composed of the expandable intravertebral implant and the supported surrounding trabeculae for low-pressure injection. In contrast to PKP, the new system maintains the cavity under the expandable intravertebral implant support, which, in theory, further reduces the possibility of cement extravasation [54]. Moreover, retaining the implant can reduce the use of bone cement, thus theoretically decreasing the occurrence of cement leakage. However, our meta-analysis indicated no differences in the amount of bone cement injected between the 2 groups, which may be attributed to the good maintenance of the cavity supported by expandable implants. Thoracic fractures are often referred to as kyphotic fractures for being associated kyphotic spinal angulation, which lead to the center of gravity being shifted more anteriorly, increasing the lever arm of the forces and the forward bending moments on the already fragile spine. These mechanical changes often result in a further compression of the fractured vertebral but also put adjacent vertebrae at a higher risk of developing new fractures [48]. Although it is still controversial whether these adjacent fractures are due to the surgical procedure or natural evolution, obtaining and maintaining a more adequate reduction via these expandable implants is essential to prevent the domino effect, which is the consecutive occurrence of OVCFs in adjacent vertebrae due to excessive anterior overload after the first uncorrected wedge-shaped vertebral body [54]. Our results also prove that expandable intravertebral implants could provide adequate stability without increasing vertebral stiffness to decrease the risk of adjacent fractures.

The limitations of our meta-analysis were as follows: First, the lack of random allocation, allocation concealment, and blinding in the no-RCTs might result selective and performance bias; second, the methods using for evaluating vertebral height change and other outcomes, surgical technologies, and instruments varied among studies, all of which increased the risk of heterogeneity; and third, given the limited number of the included studies in the analysis, the findings should be confirmed in future research with more relevant RCTs to obtain more reliable and conclusive data.

## 6. Conclusions

Based on our current evidence, third-generation PVAS provided a similar effect on pain relief and functional improvement compared with PVP/PKP at each follow-up period. However, the third-generation PVAS was more effective for local kyphosis correction, vertebral height maintenance, and with a significantly reduced risk of incidence of cement leakage and new fractures. Further high-quality RCTs are required to confirm these results.

## Figures and Tables

**Table 1:** Study characteristics of in vivo studies.

Study	Country	Study design	Sample size		Vertebral bodies (*n*)		Age (years)		Gender (M/F)		*t*-score		Interventions		NOS scores
			I	C	I	C	I	C	I	C	I	C	I	C	
Huang 2020	Taiwan, China	Retrospective	42	32	42	32	71.62 ± 9.30	73.59 ± 9.14	11/31	10/22	−2.78 ± 1.30	−2.36 ± 1.37	SpineJack	PVP	∗∗∗∗∗∗∗∗
Lin 2016	Taiwan, China	Retrospective	36	39	36	39	72.62 ± 7.5	75.73 ± 6.4	6/30	4/35	−2.076 ± 1.07	−2.062 ± 0.91	SpineJack	PVP	∗∗∗∗∗∗∗∗
Noriega 2019	Multicenter	RCT	68	73	75	80	74.4 ± 8.9	72.2 ± 10	17/51	13/60	<-2.0	<-2.0	SpineJack	PKP	
Noriega 2016	Spain	RCT	15	15	16	17	67.9 ± 4.5	68.3 ± 6.1	4/11	2/13	NR		SpineJack	PKP	
Korovessis 2013	Greece	RCT	82	86	133	122	69 ± 11	72 ± 9	26/56	23/63	NR		KIVA	PKP	
Otten 2013	Germany	Retrospective	26	26	34	34	73.6 ± 8.6	66.4 ± 8.9	6/20	11/15	NR		KIVA	PKP	∗∗∗∗∗∗
Tutton 2015	USA	RCT	144	141	177	178	76.03 ± 8.82	75.09 ± 9.62	39/105	35/106	−1.96 ± 1.58	−1.89 ± 1.49	KIVA	PKP	
Schützenberger 2018	Austria	Retrospective	36	13	36	13	68.5 ± 11.5	69.2 ± 9.7	9/27	5/8	NR		VBS	PKP	∗∗∗∗∗∗∗∗
Thaler 2013	Austria	Retrospective	27	29	55	61	66.9 (46.5–87.4)	67.9 (49.2–94.6)	4/23	3/26	NR		VBS	PVP	∗∗∗∗∗∗∗
Werner 2013	Switzerland	RCT	65		50	50	70 ± 13		40/25		NR		VBS	PKP	
Ma 2020	Mainland, China	Retrospective	15	25	15	25	76.80 ± 9.92	72.84 ± 7.53	3/12	10/15	−3.66 ± 0.76	−3.54 ± 0.62	VBS	PKP	∗∗∗∗∗∗∗

I: intervention group, C: control group, M: male, F: female, NOS: Newcastle-Ottawa scale, NR: not reported.

**Table 2:** Study characteristics of in vitro studies.

Study	Country	Study design	Specimens (*n*)	Gender (M/F)	Age (years)	Vertebral bodies (*n*)		BMD		Interventions		NOS
						I	C	I	C	I	C	
Disch 2014	Germany	Prospective	6	3/3	76.3 (63–89)	6	6	76.8 ± 10.9		VBS	PKP	∗∗∗∗∗∗∗∗∗
Rotter 2010	Germany	Prospective	4	1/3	62.3 (55–65)	12	12	0.580 ± 0.179	0.582 ± 0.195	VBS	PKP	∗∗∗∗∗∗∗∗∗
Wang 2018	China	Prospective	4	1/3	78.3 (74–81)	12	12	0.590 ± 0.115	0.582 ± 0.149	VBS	PKP	∗∗∗∗∗∗∗∗∗
Ghofrani 2010	USA	RCT	5	1/4	77 ± 10	20	19	NR		OsseoFix	PKP	
Upasani 2010	USA	RCT	4	4/0	68 ± 9	24	24	119 ± 44		OsseoFix	PKP	
Wilson 2012	USA	RCT	9	4/5	74 (58–87)	7	7	0.63 ± 0.09	0.66 ± 0.11	Kiva	PKP	
Krüger 2013	Germany	RCT	6	0/6	84.5 (79–93)	8	9	0.38 ± 0.08		SpineJack	PKP	
Krüger 2015	Germany	RCT	2	0/2	70 and 60	12	12	*T*-score (-6.8 and -6.3)		SpineJack	PKP	

I: intervention group, C: control group, M: male, F: female, NOS: Newcastle-Ottawa scale, NR: not report.

## Data Availability

The data used to support the findings of this study are available from the corresponding author upon request.

## Supplementary Materials

Supplementary Materials Search Strategies.

## References

[1] Ebeling P. R., Akesson K., Bauer D. C. (2019). The efficacy and safety of vertebral augmentation: a second ASBMR task force report. *Journal of Bone and Mineral Research*.

[2] Hopkins T. J., Eggington S., Quinn M., Nichols-Ricker C. I. (2020). Cost-Effectiveness of Balloon Kyphoplasty and Vertebroplasty Versus Conservative Medical Management in the USA. *Osteoporosis International*.

[3] Luthman S., Widén J., Borgström F. (2018). Appropriateness criteria for treatment of osteoporotic vertebral compression fractures. *Osteoporosis International*.

[4] Muijs S. P. J., Van Erkel A. R., Dijkstra P. D. S. (2011). Treatment of painful osteoporotic vertebral compression fractures. *The Journal of Bone and Joint Surgery. British volume*.

[5] Zhan Y., Jiang J., Liao H., Tan H., Yang K. (2017). Risk factors for cement leakage after vertebroplasty or kyphoplasty: a meta-analysis of published evidence. *World Neurosurgery*.

[6] Garfin S. R., Yuan H. A., Reiley M. A. (2001). New technologies in spine: kyphoplasty and vertebroplasty for the treatment of painful osteoporotic compression fractures. *Spine (Phila. Pa. 1976)*.

[7] Zhao G., Liu X., Li F. (2016). Balloon kyphoplasty versus percutaneous vertebroplasty for treatment of osteoporotic vertebral compression fractures (OVCFs). *Osteoporosis International*.

[8] Li Y.-x., Guo D.-q., Zhang S.-c. (2018). Risk factor analysis for re-collapse of cemented vertebrae after percutaneous vertebroplasty (PVP) or percutaneous kyphoplasty (PKP). *International Orthopaedics*.

[9] Heo D. H., Chin D. K., Yoon Y. S., Kuh S. U. (2009). Recollapse of previous vertebral compression fracture after percutaneous vertebroplasty. *Osteoporosis International*.

[10] Yu W. B., Jiang X. B., Liang D., Xu W. X., Ye L. Q., Wang J. (2019). Risk factors and score for recollapse of the augmented vertebrae after percutaneous vertebroplasty in osteoporotic vertebral compression fractures. *Osteoporosis International*.

[11] Wei H., Dong C., Zhu Y. (2019). Posterior fixation combined with vertebroplasty or vertebral column resection for the treatment of osteoporotic vertebral compression fractures with intravertebral cleft complicated by neurological deficits. *BioMed Research International*.

[12] Wei H., Dong C., Zhu Y., Ma H. (2020). Analysis of two minimally invasive procedures for osteoporotic vertebral compression fractures with intravertebral cleft: a systematic review and meta-analysis. *Journal of Orthopaedic Surgery and Research*.

[13] Feltes C., Fountas K. N., Machinis T. (2005). Immediate and early postoperative pain relief after kyphoplasty without significant restoration of vertebral body height in acute osteoporotic vertebral fractures. *Neurosurgical Focus*.

[14] Noriega D. C., Ramajo R. H., Lite I. S. (2016). Safety and clinical performance of kyphoplasty and SpineJack® procedures in the treatment of osteoporotic vertebral compression fractures: a pilot, monocentric, investigator-initiated study. *Osteoporosis International*.

[15] Noriega D. C., Rodrίguez-Monsalve F., Ramajo R., Sánchez-Lite I., Toribio B., Ardura F. (2019). Long-term safety and clinical performance of kyphoplasty and SpineJack® procedures in the treatment of osteoporotic vertebral compression fractures: a pilot, monocentric, investigator-initiated study. *Osteoporosis International*.

[16] Noriega D., Maestretti G., Renaud C. (2015). Clinical performance and safety of 108 SpineJack implantations: 1-year results of a prospective multicentre single-arm registry study. *BioMed Research International*.

[17] Noriega D., Krüger A., Ardura F. (2015). Clinical outcome after the use of a new craniocaudal expandable implant for vertebral compression fracture treatment: one year results from a prospective multicentric study. *BioMed Research International*.

[18] Thaler M., Lechner R., Nogler M., Gstöttner M., Bach C. (2013). Surgical procedure and initial radiographic results of a new augmentation technique for vertebral compression fractures. *European Spine Journal*.

[19] Schützenberger S., Schwarz S. M., Greiner L. (2018). Is vertebral body stenting in combination with CaP cement superior to kyphoplasty?. *European Spine Journal*.

[20] Werner C. M. L., Osterhoff G., Schlickeiser J. (2013). Vertebral body stenting versus kyphoplasty for the treatment of osteoporotic vertebral compression fractures. *The Journal of Bone and Joint Surgery*.

[21] Upasani V. V., Robertson C., Lee D., Tomlinson T., Mahar A. T. (2010). Biomechanical comparison of kyphoplasty versus a titanium mesh implant with cement for stabilization of vertebral compression fractures. *Spine (Phila. Pa. 1976)*.

[22] Ghofrani H., Nunn T., Robertson C., Mahar A., Lee Y., Garfin S. (2010). An evaluation of fracture stabilization comparing kyphoplasty and titanium mesh repair techniques for vertebral compression fractures: is bone cement necessary?. *spine (Phila. Pa. 1976)*.

[23] Tutton S. M., Pflugmacher R., Davidian M., Beall D. P., Facchini F. R., Garfin S. R. (2015). KAST study: the Kiva system as a vertebral augmentation treatment-a safety and effectiveness trial: a randomized, noninferiority trial comparing the Kiva system with balloon kyphoplasty in treatment of osteoporotic vertebral compression fractures. *Spine (Phila. Pa. 1976)*.

[24] Otten L. A., Bornemnn R., Jansen T. R. (2013). Comparison of balloon kyphoplasty with the new Kiva® VCF system for the treatment of vertebral compression fractures. *Pain Physician*.

[25] Korovessis P., Vardakastanis K., Repantis T., Vitsas V. (2013). Balloon kyphoplasty versus KIVA vertebral augmentation—comparison of 2 techniques for osteoporotic vertebral body fractures. *Spine (Phila. Pa. 1976)*.

[26] Noriega D., Marcia S., Theumann N. (2019). A prospective, international, randomized, noninferiority study comparing an implantable titanium vertebral augmentation device versus balloon kyphoplasty in the reduction of vertebral compression fractures (SAKOS study). *The Spine Journal*.

[27] Moher D., Liberati A., Tetzlaff J., Altman D. G., for the PRISMA Group (2009). Preferred reporting items for systematic reviews and meta-analyses: the PRISMA statement. *BMJ*.

[28] Higgins J., Thomas J., Chandler J. (2019). Cochrane Handbook for Systematic Reviews of Interventions Version 6.0 (Updated July 2019). *Cochrane*.

[29] Higgins J. P. T., Altman D. G., Gøtzsche P. C. (2011). The Cochrane collaboration’s tool for assessing risk of bias in randomised trials. *BMJ*.

[30] Stang A. (2010). Critical evaluation of the Newcastle-Ottawa scale for the assessment of the quality of nonrandomized studies in meta-analyses. *European Journal of Epidemiology*.

[31] Higgins J. P. T., Thompson S. G. (2002). Quantifying heterogeneity in a meta-analysis. *Statistics in Medicine*.

[32] Ma Y., Zhang S., Yuan K. (2020). Therapeutic effects of percutaneous vertebroplasty and kyphoplasty on osteoporotic vertebral compression fracture. *Chinese Journal of Tissue Engineering Research*.

[33] Huang C.-C., Tai S.-H., Lai C.-H., Lee E.-J. (2020). Comparison of an intravertebral reduction device and percutaneous vertebroplasty for anatomical reduction with single-level vertebral compression fractures. *Formosan Journal of Surgery*.

[34] Lin J.-H., Wang S.-H., Lin E.-Y., Chiang Y.-H. (2016). Better height restoration, greater kyphosis correction, and fewer refractures of cemented vertebrae by using an intravertebral reduction device: a 1-year follow-up study. *World Neurosurgery*.

[35] Krüger A., Oberkircher L., Figiel J. (2015). Height restoration of osteoporotic vertebral compression fractures using different intravertebral reduction devices: a cadaveric study. *The Spine Journal*.

[36] Rotter R., Martin H., Fuerderer S. (2010). Vertebral body stenting: a new method for vertebral augmentation versus kyphoplasty. *European Spine Journal*.

[37] Wilson D. C., Connolly R. J., Zhu Q. (2012). An ex vivo biomechanical comparison of a novel vertebral compression fracture treatment system to kyphoplasty. *Clinical biomechanics*.

[38] Krüger A., Baroud G., Noriega D. (2013). Height restoration and maintenance after treating unstable osteoporotic vertebral compression fractures by cement augmentation is dependent on the cement volume used. *Clinical biomechanics*.

[39] Disch A. C., Schmoelz W. (2014). Cement augmentation in a thoracolumbar fracture model: reduction and stability after balloon kyphoplasty versus vertebral body stenting. *Spine (Phila. Pa. 1976)*.

[40] Wang D., Zheng S., Liu A. (2018). The role of minimally invasive vertebral body stent on reduction of the deflation effect after kyphoplasty. *Spine (Phila. Pa. 1976)*.

[41] Lange A., Zeidler J., Braun S. (2014). One-year disease-related health care costs of incident vertebral fractures in osteoporotic patients. *Osteoporosis International*.

[42] Chen A. T., Cohen D. B., Skolasky R. L. (2013). Impact of nonoperative treatment, vertebroplasty, and kyphoplasty on survival and morbidity after vertebral compression fracture in the Medicare population. *The Journal of Bone and Joint Surgery*.

[43] Zampini J. M., White A. P., McGuire K. J. (2010). Comparison of 5766 vertebral compression fractures treated with or without kyphoplasty. *Clinical Orthopaedics and Related Research*.

[44] Kallmes D. F., Comstock B. A., Heagerty P. J. (2009). A randomized trial of vertebroplasty for osteoporotic spinal fractures. *The New England Journal of Medicine*.

[45] Buchbinder R., Osborne R. H., Ebeling P. R. (2009). A randomized trial of vertebroplasty for painful osteoporotic vertebral fractures. *The New England Journal of Medicine*.

[46] Chang M., Zhang C., Shi J. (2021). Comparison between 7 osteoporotic vertebral compression fractures treatments: systematic review and network meta-analysis. *World Neurosurgery*.

[47] Long Y., Yi W., Yang D. (2020). Advances in vertebral augmentation systems for osteoporotic vertebral compression fractures. *Pain Research & Management*.

[48] Manz D., Georgy M., Beall D. P., Baroud G., Georgy B. A., Muto M. (2020). Vertebral augmentation with spinal implants: third-generation vertebroplasty. *Neuroradiology*.

[49] Vanni D., Galzio R., Kazakova A. (2016). Third-generation percutaneous vertebral augmentation systems. *Journal of Spine Surgery*.

[50] El-Fiki M. (2016). Vertebroplasty, kyphoplasty, lordoplasty, expandable devices, and current treatment of painful osteoporotic vertebral fractures. *World Neurosurgery*.

[51] Taylor R. S., Fritzell P., Taylor R. J. (2007). Balloon kyphoplasty in the management of vertebral compression fractures: an updated systematic review and meta-analysis. *European Spine Journal*.

[52] Gill J. B., Kuper M., Chin P. C., Zhang Y., Schutt R. (2007). Comparing pain reduction following kyphoplasty and vertebroplasty for osteoporotic vertebral compression fractures: meta-analysis. *Pain Physician*.

[53] Chang X., Lv Y.-F., Chen B. (2015). Vertebroplasty versus kyphoplasty in osteoporotic vertebral compression fracture: a meta-analysis of prospective comparative studies. *International Orthopaedics*.

[54] Moura D. L., Gabriel J. P. (2021). Expandable intravertebral implants: a narrative review on the concept, biomechanics, and outcomes in traumatology. *Cureus*.

