# Supplementary material for: Therapeutic Efficacy of Third-Generation Percutaneous Vertebral Augmentation System (PVAS) in Osteoporotic Vertebral Compression Fractures (OVCFs): A Systematic Review and Meta-analysis
Source: Biomed Res Int. 2022 May 7;2022:9637831. doi: 10.1155/2022/9637831 (PMC9107362; doi:10.1155/2022/9637831)
Supplement: Supplementary Materials — Search Strategies. [file 9637831.f1.docx]

Search Strategies

Pubmed:

| Search number | Query | Sort By | Filters | Search Details | Results |
| --- | --- | --- | --- | --- | --- |
| 18 | (((((Spinal Fracture*) OR (thoracic fracture*)) OR (lumbar fracture*)) OR (vertebral fracture*)) OR ("Spinal Fractures"[Mesh])) AND ((((((KIVA) OR (spinejack)) OR (vertebral body stent*)) OR (Stentoplasty)) OR (VBS)) OR (OsseoFix)) | | | ((("spinal"[All Fields] OR "spinalization"[All Fields] OR "spinalized"[All Fields] OR "spinally"[All Fields] OR "spinals"[All Fields]) AND "fracture*"[All Fields]) OR (("thoracal"[All Fields] OR "thoracical"[All Fields] OR "thorax"[MeSH Terms] OR "thorax"[All Fields] OR "thoracic"[All Fields] OR "thoracics"[All Fields]) AND "fracture*"[All Fields]) OR (("lumbarised"[All Fields] OR "lumbarization"[All Fields] OR "lumbarized"[All Fields] OR "lumbars"[All Fields] OR "lumbosacral region"[MeSH Terms] OR ("lumbosacral"[All Fields] AND "region"[All Fields]) OR "lumbosacral region"[All Fields] OR "lumbar"[All Fields]) AND "fracture*"[All Fields]) OR (("spine"[MeSH Terms] OR "spine"[All Fields] OR "vertebral"[All Fields] OR "vertebrals"[All Fields]) AND "fracture*"[All Fields]) OR "Spinal Fractures"[MeSH Terms]) AND ("kiva"[Journal] OR "kiva"[All Fields] OR "spinejack"[All Fields] OR (("vertebral body"[MeSH Terms] OR ("vertebral"[All Fields] AND "body"[All Fields]) OR "vertebral body"[All Fields]) AND "stent*"[All Fields]) OR "Stentoplasty"[All Fields] OR "VBS"[All Fields] OR "OsseoFix"[All Fields]) | 128 |
| 17 | ((((Spinal Fracture*) OR (thoracic fracture*)) OR (lumbar fracture*)) OR (vertebral fracture*)) OR ("Spinal Fractures"[Mesh]) | | | (("spinal"[All Fields] OR "spinalization"[All Fields] OR "spinalized"[All Fields] OR "spinally"[All Fields] OR "spinals"[All Fields]) AND "fracture*"[All Fields]) OR (("thoracal"[All Fields] OR "thoracical"[All Fields] OR "thorax"[MeSH Terms] OR "thorax"[All Fields] OR "thoracic"[All Fields] OR "thoracics"[All Fields]) AND "fracture*"[All Fields]) OR (("lumbarised"[All Fields] OR "lumbarization"[All Fields] OR "lumbarized"[All Fields] OR "lumbars"[All Fields] OR "lumbosacral region"[MeSH Terms] OR ("lumbosacral"[All Fields] AND "region"[All Fields]) OR "lumbosacral region"[All Fields] OR "lumbar"[All Fields]) AND "fracture*"[All Fields]) OR (("spine"[MeSH Terms] OR "spine"[All Fields] OR "vertebral"[All Fields] OR "vertebrals"[All Fields]) AND "fracture*"[All Fields]) OR "Spinal Fractures"[MeSH Terms] | 50,641 |
| 16 | (((Spinal Fracture*) OR (thoracic fracture*)) OR (lumbar fracture*)) OR (vertebral fracture*) | | | (("spinal"[All Fields] OR "spinalization"[All Fields] OR "spinalized"[All Fields] OR "spinally"[All Fields] OR "spinals"[All Fields]) AND "fracture*"[All Fields]) OR (("thoracal"[All Fields] OR "thoracical"[All Fields] OR "thorax"[MeSH Terms] OR "thorax"[All Fields] OR "thoracic"[All Fields] OR "thoracics"[All Fields]) AND "fracture*"[All Fields]) OR (("lumbarised"[All Fields] OR "lumbarization"[All Fields] OR "lumbarized"[All Fields] OR "lumbars"[All Fields] OR "lumbosacral region"[MeSH Terms] OR ("lumbosacral"[All Fields] AND "region"[All Fields]) OR "lumbosacral region"[All Fields] OR "lumbar"[All Fields]) AND "fracture*"[All Fields]) OR (("spine"[MeSH Terms] OR "spine"[All Fields] OR "vertebral"[All Fields] OR "vertebrals"[All Fields]) AND "fracture*"[All Fields]) | 50,641 |
| 15 | "Spinal Fractures"[Mesh] | Most Recent | | "Spinal Fractures"[MeSH Terms] | 15,197 |
| 12 | (((((KIVA) OR (spinejack)) OR (vertebral body stent*)) OR (Stentoplasty)) OR (VBS)) OR (OsseoFix) | | | "kiva"[Journal] OR "kiva"[All Fields] OR "spinejack"[All Fields] OR (("vertebral body"[MeSH Terms] OR ("vertebral"[All Fields] AND "body"[All Fields]) OR "vertebral body"[All Fields]) AND "stent*"[All Fields]) OR "Stentoplasty"[All Fields] OR "VBS"[All Fields] OR "OsseoFix"[All Fields] | 794 |

Embase:

No. Query Results Results

#14. #6 AND #13 172

#13. #7 OR #8 OR #9 OR #10 OR #11 OR #12 1,175

#12. 'osseofix' 26

#11. 'vbs' 852

#10. 'stentoplasty' 31

#9. 'vertebral body sent*'

#8. 'spinejack' 56

#7. 'kiva' 238

#6. #1 OR #2 OR #3 OR #4 OR #5 40,373

#5. 'vertebral fracture*':ab,ti 13,542

#4. 'lumbar fracture*':ab,ti 543

#3. 'thoracic fracture*':ab,ti 180

#2. 'spinal fracture*':ab,ti 2,129

#1. 'spine fracture'/exp 37,471

Cochrane Library:

ID Search Hits

#1 MeSH descriptor: [Spinal Fractures] explode all trees 718

#2 (Spinal Fracture*):ti,ab,kw OR (thoracic fracture*):ti,ab,kw OR (lumbar fracture*):ti,ab,kw OR (vertebral fracture*):ti,ab,kw (Word variations have been searched) 4933

#3 #1 OR #2 4933

#4 (kiva) OR (spinejack) OR (vertebral body sent*) OR (Stentoplasty) OR (VBS) OR (OsseoFix) (Word variations have been searched) 145

#5 #3 AND #4 37
